# Development of a nomogram for predicting postoperative Hirschsprung-associated enterocolitis

**DOI:** 10.3389/fped.2025.1640331

**Published:** 2025-09-16

**Authors:** Weijun Zheng, Weiming Chen, Fei Chen, Yunjin Wang, Lanxin Zhu, Mingkun Liu

**Affiliations:** ^1^ Department of Pediatric Surgery, Fujian Children’s Hospital (Fujian Branch of Shanghai Children’s Medical Center), College of Clinical Medicine for Obstetrics & Gynecology and Pediatrics, Fujian Medical University, Fuzhou, China; ^2^ Department of Pediatric Surgery, Fujian Maternity and Child Health Hospital, College of Clinical Medicine for Obstetrics & Gynecology and Pediatrics, Fujian Medical University, Fuzhou, China

**Keywords:** nomogram, Hirschsprung-associated enterocolitis, risk factors, children, predicting

## Abstract

**Objectives:**

To analyze the risk factors for postoperative Hirschsprung-associated enterocolitis (HAEC) and establish a nomogram to predict the incidence of HAEC.

**Methods:**

All patients with Hirschsprung disease who underwent definitive surgery at Fujian Provincial Children's Hospital from January 2015 to December 2023 were included in the study. Univariate and multivariate logistic regression were used to analyze the influencing factors of chylous ascites and a nomogram was established. The predictive performance of the nomogram was evaluated using receiver operating characteristic (ROC) curves, calibration plots, and decision curve analysis (DCA) curves.

**Results:**

Of the included 204 patients, 53 patients (25.9%) experienced postoperative HAEC. Preoperative HAEC, the type of HSCR (long-segment or total colonic aganglionosis), no-preoperative bowel preparation, and anastomotic leaks or strictures were considered important risk factors. The area under the ROC curve of the model is 0.79, the nomogram has great discriminative ability, calibration and significant clinical utility.

**Conclusion:**

We found a nomogram for predicting the postoperative HAEC. It can be used as a reference for risk assessment and early detection of postoperative HAEC.

## Introduction

Hirschsprung disease (HSCR) is characterized by the complete absence of neuronal ganglion cells in a segment of the intestinal tract, most commonly in the large intestine (1). At present, surgery remains the primary effective treatment option for patients with HSCR, with the aganglionic section being removed. Hirschsprung-associated enterocolitis (HAEC) is a prevalent and severe complication after radical surgery for HSCR, which can lead to bowel obstruction and sepsis, with a high mortality rate (2). The typical clinical features of HAEC include explosive diarrhea, abdominal distension, fever, lethargy and vomiting. However, these symptoms are non-specific and can be similar to those of other diseases like gastroenteritis, which makes it difficult to identify HAEC in clinical practice and may lead to delayed treatment (3).

Although all patients diagnosed with HSCR are theoretically at risk of developing HAEC, a variety of factors have been identified that contribute to an elevated risk of this complication. These include family history of HSCR, trisomy-21, long-segment aganglionosis, delayed diagnosis, anastomotic leaks or strictures and prior episodes of HAEC (4–12). In addition, intestinal dysbiosis, macrophage infiltration, and neuroimmune abnormalities collectively promote the occurrence of HAEC (5, 13–15). Despite the identification of multiplerisk factors for HAEC, a predictive model for postoperative HAEC has not yet been established. Accordingly, we developed the first nomogram to predict the incidence of HAEC.

## Materials and methods

### Study design and patients

This study was approved by the Ethics Committee of Fujian Children's Hospital. Patients with confirmed HSCR were conducted at Fujian Children's Hospital, from January 2015 to December 2023. Inclusion criteria: (1) Patients who received surgical treatment at the Fujian Children's Hospital. (2) Patients diagnosed with HSCR based on postoperative pathological results. Exclusion criteria: (1) Postoperative loss of visit. (2) Combination with other congenital anomalies. Diagnostic criteria for HAEC: A score of ≥4 was included in the diagnosis of HAEC according to the scoring system proposed by Pastor (16). The HAEC scoring criteria developed include 18 indicators, covering aspects such as medical history, physical examination, radiological examination, and laboratory examination (17).

### Variables

Variables analyzed as risk factors for HAEC included weight, age, sex, enterostomy, standardized anal dilatation, preoperative bowel preparation, preoperative HAEC, type of surgery, type of HSCR, anastomotic leaks or strictures, preoperative hemoglobin concentration and preoperative serum albumin concentrations.

### Statistical analysis

Statistical analysis was performed using IBM SPSS (23.0) and R software (4.4.1). Continuous variables are presented as the mean ± standard deviation (SD) and were compared using the Mann–Whitney *U*-test. The categorical data were presented in count and percentage. The continuous data were presented in mean with SD for normally distributed data and the median with interquartile range for non-normal data distribution. Univariate and multivariate factors were analyzed by logistic regressions. When the *P*-value <0.05, the differences were determined of statistical significance. Nomograms are constructed based on the independent prognostic factors. The receiver operating characteristic (ROC) curves are generated using the data from the training and validation sets, and the area under the curve (AUC) is determined to evaluate the discriminatory capacity of the model. Calibration curves are plotted to examine the accuracy of the model, while decision curve analysis (DCA) is utilized to assess the clinical practicality of the model.

## Result

### Clinical characteristics and risk factors of postoperative HAEC

A total of 204 (165 males and 39 females) patients with HSCR, among whom 53 (43 males and 10 females) with HAEC (HAEC group) and 151 (122 males and 29 females) did not with HAEC (non-HAEC group). After univariate logistic regression analysis of the clinical data of the two groups, we found patients who underwent preoperative bowel preparation [HAEC (43.34%) vs. non-HAEC (67.55%), *P* = 0.002] or standardized anal dilatation [HAEC (20.75%) vs. non-HAEC (38.41%), *P* = 0.019] were significantly fewer in the postoperative HAEC group compared with the non-HAEC group. The HAEC group had a higher frequency of enterostomy [HAEC (18.87%) vs. non-HAEC (8.61%), *P* = 0.042] and preoperative HAEC [HAEC (49.06%) vs. non-HAEC (22.52%), *P* < 0.01]. In the HAEC group, the long-segment and the total colonic aganglionosis were significantly more than the non-HAEC group. Patients with postoperative anastomotic leaks or strictures [HAEC (18.87%) vs. non-HAEC (3.97%), *P* = 0.002] had a higher incidence of HAEC (Table 1).

**Table 1:** Baseline characteristics of patients in Non-HAEC and HAEC groups (*n* = 204).

Variables	Total (*n* = 204)	Non-HAEC No (*n* = 151)	HAEC Yes (*n* = 53)	Statistic	*P*
Weight (kg), M (Q₁, Q₃)	8.00 (6.79, 9.12)	8.00 (7.00, 9.30)	7.80 (6.60, 9.00)	Z = −1.19	0.234
Sex, *n* (%)					
Female	39 (19.12)	29 (19.21)	10 (18.87)	χ^2^ = 0.00	0.957
Male	165 (80.88)	122 (80.79)	43 (81.13)		
Age (month), *n* (%)					
<6	100 (49.02)	71 (47.02)	29 (54.72)	χ^2^ = 2.85	0.241
6–12	65 (31.86)	47 (31.13)	18 (33.96)		
≥12	39 (19.12)	33 (21.85)	6 (11.32)		
Enterostomy, *n* (%)					
No	181 (88.73)	138 (91.39)	43 (81.13)	χ^2^ = 4.13	0.042
Yes	23 (11.27)	13 (8.61)	10 (18.87)		
Standardized anal dilatation, *n* (%)					
No	135 (66.18)	93 (61.59)	42 (79.25)	χ^2^ = 5.46	0.019
Yes	69 (33.82)	58 (38.41)	11 (20.75)		
Preoperative bowel preparation, *n* (%)					
No	79 (38.73)	49 (32.45)	30 (56.60)	χ^2^ = 9.65	0.002
Yes	125 (61.27)	102 (67.55)	23 (43.40)		
Preoperative HAEC, *n* (%)					
No	144 (70.59)	117 (77.48)	27 (50.94)	χ^2^ = 13.31	<.001
Yes	60 (29.41)	34 (22.52)	26 (49.06)		
Laparoscopic, *n* (%)					
No	44 (21.57)	33 (21.85)	11 (20.75)	χ^2^ = 0.03	0.867
Yes	160 (78.43)	118 (78.15)	42 (79.25)		
Type of HSCR, *n* (%)					
Short-segment	74 (36.27)	63 (41.72)	11 (20.75)	χ^2^ = 14.15	0.003
Typical-segment	84 (41.18)	63 (41.72)	21 (39.62)		
Long-segment	38 (18.63)	21 (13.91)	17 (32.08)		
Total colonic	8 (3.92)	4 (2.65)	4 (7.55)		
Anastomotic leaks or strictures, *n* (%)					
No	188 (92.16)	145 (96.03)	43 (81.13)	χ^2^ = 10.07	0.002
Yes	16 (7.84)	6 (3.97)	10 (18.87)		
Preoperative hemoglobin concentration (g/L), Mean ± SD	110.89 ± 11.81	110.87 ± 12.22	110.94 ± 10.68	t = −0.04	0.971
Preoperative serum albumin concentrations (g/L), M (Q₁, Q₃)	40.00 (39.00, 43.00)	40.00 (39.00, 43.00)	40.00 (39.00, 42.00)	Z = −0.11	0.909

*t*, *t*-test; Z, Mann–Whitney test; χ^2^, Chi-square test; SD, standard deviation; M, Median; Q₁, 1st Quartile; Q₃, 3st Quartile.

### Multivariate analysis of predictive factors for postoperative HAEC

We incorporated all predictors with statistical significance in univariate logistic regression analysis into the multivariate logistic regression analysis. Among them, preoperative HAEC, long-segment or total colonic aganglionosis, and anastomotic leaks or strictures are independent predictive factors influencing postoperative HAEC (*P* < 0.05). Preoperative bowel preparation is a protective factor for postoperative HAEC (*P* < 0.05) (Table 2).

**Table 2:** Univariate and multivariate logistic regression analyses of risk factors for HAEC.

Variables	Univariate	Multivariate				
	*P*	*β*	S.E	*Z*	*P*	OR (95% CI)
Enterostomy	0.047	−0.95	0.71	−1.35	0.177	0.38 (0.10–1.54)
Standardizedanal dilatation	0.022	−0.73	0.43	−1.70	0.089	0.48 (0.21–1.12)
Preoperative bowel preparation	0.002	−1.43	0.42	−3.39	<.001	0.24 (0.10–0.55)
Preoperative HAEC	<.001	1.05	0.39	2.68	0.007	2.87 (1.33–6.21)
Type of HSCR						
Long-segment						1.00
Short-segment	<.001	−1.72	0.57	−3.03	0.002	0.18 (0.06–0.54)
Total colonic aganglionosis	0.786	0.06	0.94	0.07	0.948	1.06 (0.17–6.67)
Typical-segment	0.031	−0.91	0.49	−1.85	0.064	0.40 (0.16–1.05)
Anastomotic leaks and strictures	0.002	1.84	0.61	3.03	0.002	6.32 (1.92–20.84)

OR, odds ratio; CI, confidence interval.

### Nomogram for predicting postoperative HAEC

**Figure 1:**
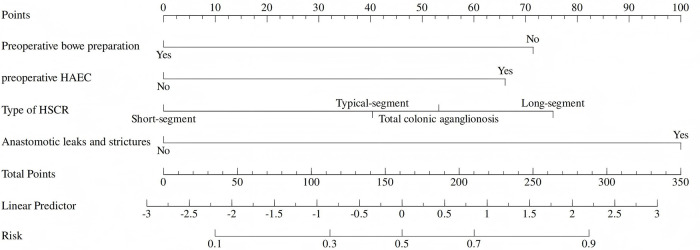
Nomogram for predicting postoperative HAEC. (The nomogram to predict the postoperative HAEC was created based on four independent risk factors, including postoperative HAEC, long-segment or total colonic aganglionosis, anastomotic leaks or strictures, and preoperative bowel preparation).

**Figure 2:**
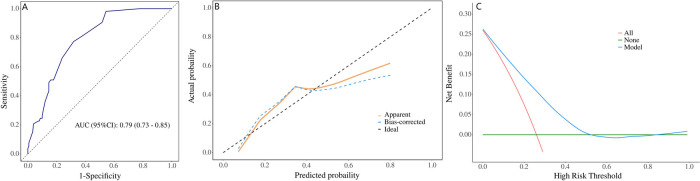
Validation of the nomogram. [**(A)** ROC curve for the nomogram, AUC = 0.79 (95% CI: 0.73–0.85). **(B)** Nomogram calibration curve. **(C)** DCA curve of nomogram. ROC, receiver operating characteristic; AUC, area under the receiver operating characteristic curve; DCA, decision curve analysis].

The independent factors above-mentioned including preoperative HAEC, long-segment or total colonic aganglionosis, anastomotic leaks or strictures, and preoperative bowel preparation are used to construct a nomogram (Figure 1). The AUC of the ROC curve of the nomogram is 0.79, the sensitivity is 0.68 (95% CI: 0.61–076), and the specificity is 0.77 (95% CI: 0.66–0.89). Indicating that the nomogram has a great performance in predicting postoperative HAEC. Calibration curves shown that the calibration curve is relatively evenly distributed around the ideal line, indicating great consistency between the nomogram's predictions and the actual results. Decision plots (DCA) demonstrate that our predictive model has great clinical applicability (Figure 2).

## Discussion

HAEC is a serious complication and significant cause of mortality among patients after operation for HSCR (18). Previous studies reported an incidence of postoperative HAEC ranging from 17% to 50% (3, 19, 20). In our study, the prevalence of HAEC was 26%, which is consistent with previous reports. The etiology and pathogenesis of HAEC are still unclear, the diagnosis of HAEC relies on clinical manifestations and symptoms which are often non-specific, making it difficult to establish a definitive diagnosis in many patients (21). In 2009, Pastor (16) proposed a scoring system for the clinical diagnosis of HAEC, which combines clinical, laboratory, and radiological parameters. They use a HAEC score of 10 or greater indicated diagnosis of HAEC. In 2018, Frykman (17) conducted an evaluation involving 116 children diagnosed with HSCR and posited that when the HAEC score was set at 4 or greater, the sum of sensitivity (83.7%) and specificity (98.6%) was optimal. Despite the HAEC scoring system's recent surge in popularity as a diagnostic tool, it is imperative to acknowledge its primary function as a diagnostic instrument rather than a predictive one. The purpose of this study is to construct a prediction model of HAEC for early detection of HAEC.

In recent years, research has identified a variety of risk factors associated with the development of HAEC. However, no studies have demonstrated a significant correlation between factors such as gender, age, preoperative hypoalbuminemia, and surgical method and the occurrence of HAEC (22, 23). In this study, we found the risk factors for HAEC include postoperative HAEC, long-segment or total colonic aganglionosis, and anastomotic leaks or strictures. Preoperative bowel preparation is a protective factor for postoperative HAEC.

We defined aganglionosis from the sigmoid colon onward as long segment and aganglionosis from the whole colon as total colonic aganglionosis (1). Previous studies have found that long-segment and total colonic aganglionosis are risk factors for HAEC (5, 24–26). A widely acknowledged predictor, long-segment have been proved to increase risk of postoperative HAEC by several studies (5). In terms of pathogenesis, long-segment and total colonic aganglionosis may lead to more severe intestinal motility and stasis due to a wider range of enteric nervous system dysfunction, which may lead to an increased risk of bacterial retention, overgrowth and translocation. This series of changes may contribute to the development of HAEC. Preoperative HAEC indicates that the intestine is already in an inflammatory state, and the intestinal barrier function, microbiota balance and immune state may have been impaired. In children with preoperative HAEC, there is an elevation in the quantity of Candida within the feces, concurrent with a reduction in the numbers of Malassezia and yeast (13). All of these factors augment the probability of postoperative recurrence of HAEC, thereby subjecting the postoperative intestine to an elevated risk of inflammation. The history of preoperative HAEC may persistently influence the incidence of postoperative HAEC (11). Intestinal flora translocation, macrophage phenotypic imbalance, and neuroimmune regulatory dysfunction collectively contribute to the pathogenesis of HAEC via intricate interactions. Postoperative intestinal motility disorders in HSCR lead to fecal stasis, which triggers dysbiosis, impairs the integrity of the intestinal barrier, and induces intestinal inflammation (13). The infiltration of M1-type macrophages increases in the lesioned intestinal segments (14). Neuroimmune regulatory disorders further amplify the inflammatory effect (15).

Anastomotic stenosis represents the primary etiological factor for intestinal obstruction subsequent to HSCR. Mechanical intestinal obstruction resulting from anastomotic complications will remarkably augment the risk of postoperative HAEC (7). Anastomotic leaks significantly elevates the risk and severity of postoperative HAEC through disrupting the intestinal barrier, facilitating bacterial translocation, and triggering chronic inflammation, thus emerging as a critical adverse prognostic factor warranting focused clinical surveillance (11, 27). Preoperative rectal irrigation preparation to alleviate mechanical intestinal obstruction can decrease the incidence of HAEC (28). In 2017, a guideline in the United States proposed that routine rectal washouts should be considered a preventative effective measure for HAEC (3). For prophylactic prevention of HAEC, some have advocated for routine rectal washouts in select populations (29). In addition, routine anal dilation within 2 weeks postoperatively and continue for three months can decrease the incidence of HAEC (30). However, there are also questions about the necessity of daily anal dilation. A study found daily dilation by family members to have similar efficacy to weekly dilations by medical staff with similar rates of HAEC (31). Antibiotics are a mainstay of treatment for HAEC, early administration of antibiotics to prevent disease progression should be considered in clinical suspicion of HAEC. Metronidazole is the most commonly used agent and should be instituted even if patients are manifesting mild symptoms (32). A prospective randomized trial found that 4 weeks of probiotic therapy reduced the incidence and severity of HAEC, but more research is needed to further determine its effectiveness in preventing HAEC (33).

Nomogram is a simple visual clinical prediction model. In this study, the AUC of the ROC curve is 0.79, indicating that the nomogram has a great performance in predicting postoperative HAEC. The calibration curve is relatively evenly distributed around the ideal line, indicating great consistency between the nomogram's predictions and the actual results. However, this study has several limitations. First, this study was of a small sample size and was conducted at a single- centre. Second, Most of the risk factors such as preoperative bowel preparation and preoperative HAEC, were hypothetical and have not been confirmed on a scientific basis. We expect to evaluate the performance of this nomogram through prospective external validation and provide treatment guidance for patients with HAEC.

## Conclusion

In this study, the risk factors for postoperative HAEC were selected by multivariate analysis, and a nomogram was constructed to predict the possibility of HAEC. The nomogram has good predictive ability for HAEC, and may provide a reference for the need of postoperative preventive measures.

## Data Availability

The original contributions presented in the study are included in the article/Supplementary Material, further inquiries can be directed to the corresponding author.
